# Supplementary material for: Long-term time-lapse microscopy of C. elegans post-embryonic development
Source: Nat Commun. 2016 Aug 25;7:12500. doi: 10.1038/ncomms12500 (PMC5512614; doi:10.1038/ncomms12500)
Supplement: Supplementary Information — Supplementary Figures 1-6 [file ncomms12500-s1.pdf]

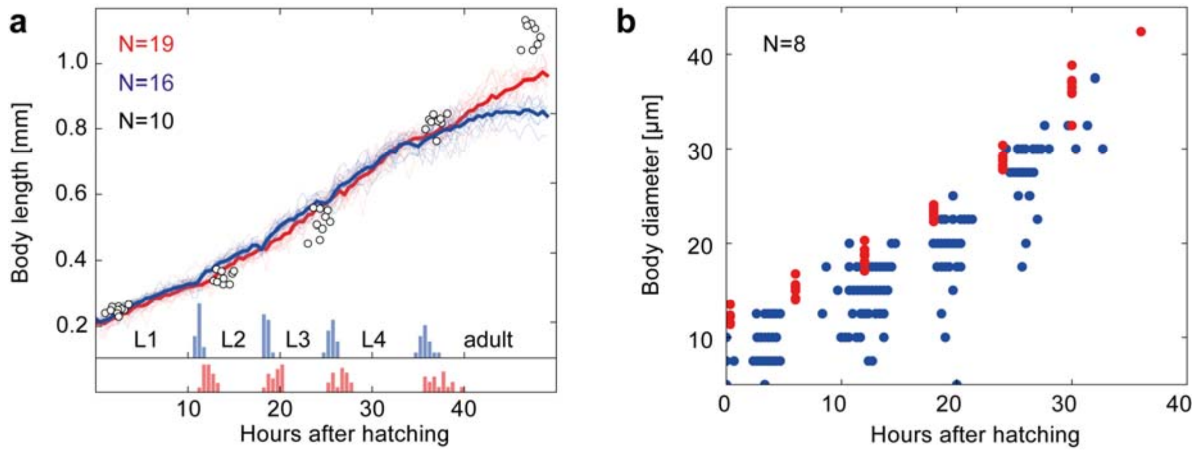

Supplementary Figure 1. Dependence of growth and developmental timing on microchamber dimensions.

(a) Body length (thin lines for individual animals and thick lines for population averages) and fraction of animals in ecdysis (bars) as a function of time for animals grown in 250µmx250µmx20µm (blue) and 290µmx290µmx25µm (red) microchambers. White markers represent body length of single animals grown on standard agar plates. Time of ecdysis is defined by the appearance of a newly shed cuticle. For animals on agar plates, we singled embryos onto 3.5cm NGM agar plates spotted with *E. coli* OP50 and detected hatching time by visual inspection. Every 12 hours, we manually imaged animals with 40X or 10X objectives and measured body length. We observed only limited difference in dynamics between growth in the two different microchambers and on agar plates, for all larval stages. The slowdown of growth upon reaching adulthood observed in microchambers, as compared to agar plates, visually coincides with the depletion of food. Finally, the variability in developmental dynamics is not reduced in the larger microchambers, as compared to the standard microchambers.
(b) Measure of the animal diameter in the vertical/Z direction(blue) and horizontal/XY direction (red). To quantify deformation of animals in 250µmx250µmx20µm microchambers, we used the strain *wIs51[SCM::GFP]* (see Seam cell lineage section in the main text) and measured the distance between left-right pairs of the V3 seam cell as a measure of the dimension of the body in the Z direction. We quantified XY dimensions by measuring the dorsal-ventral distance in the center of the body in the transmitted light images. We find that for animals older than 20 hours, the average XY dimension (32.5µm) is only 20% larger than the Z dimension (25.8µm). This shows that the hydrogel deforms to accommodate the larger (>20µm) body width of older larvae, with only modest compression of the body in the vertical direction.

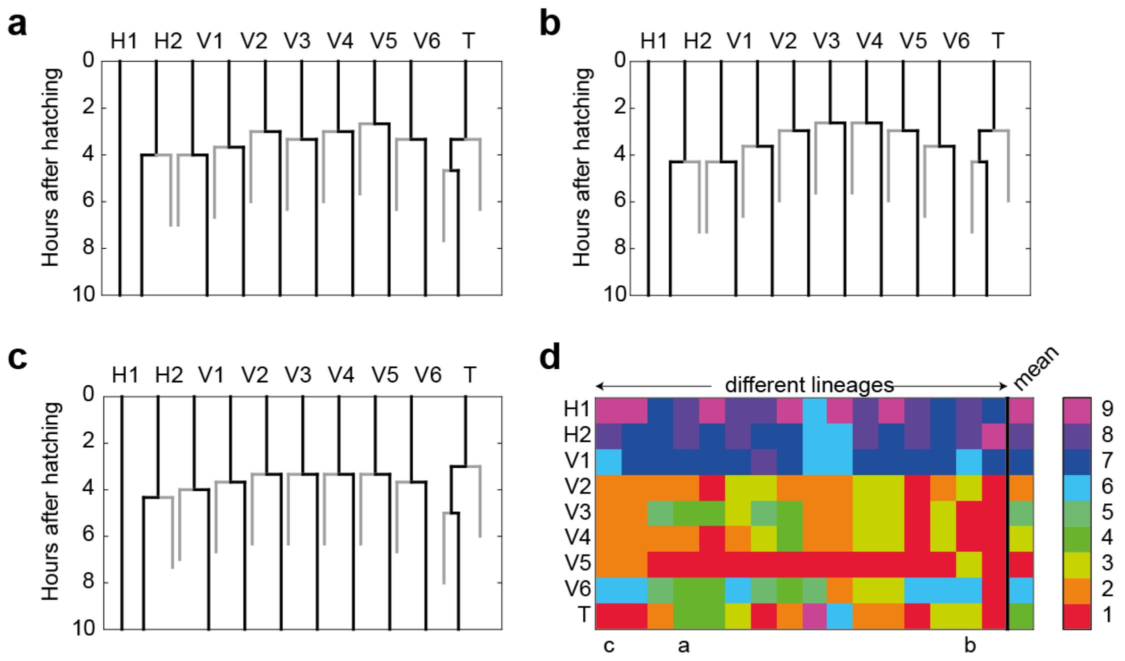

Supplementary Figure 2. Variability in the sequence of seam cell divisions.

(a), **(b)**, **(c)** Examples of animals with a typical (a) and atypical sequence (b,c) of seam cell divisions in the L1 larval stage. In Panel (a), V5 divides first. In Panel (b), V3 and V4 divide simultaneously 20 minutes before V5. In Panel (c), T divides first. **(d)** Colormap showing the sequence of seam cell divisions in the L1 larval stage, for two sides (left/right) in N=8 animals. Cells are color coded according to division sequence. The last column represents the mean division sequence. The columns labeled with (a), (b) or (c) correspond to the lineages shown in the corresponding panels.

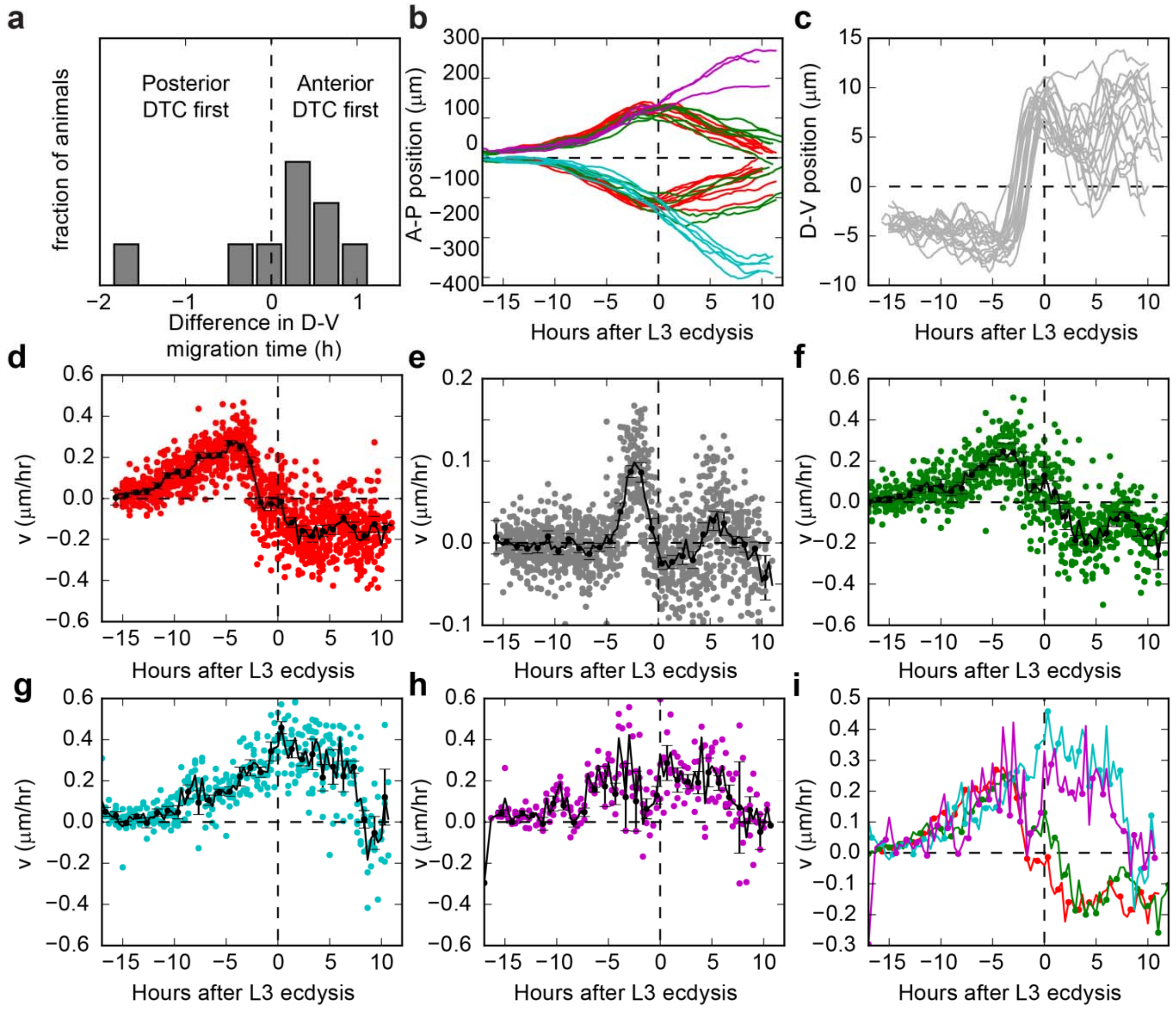

Supplementary Figure 3. Migration order and speed in distal tip cell migration.

(a) Difference in hours between D-V migration of the anterior and posterior distal tip cell (DTC), as measured by the time at which each DTC crosses the central body axis. **(b)** Sliding average of the A-P position of each DTC, with a window size of 80 min, for wild-type *qIs56* animals (red lines) and *unc-5; qIs56* mutants. For analysis of the *unc-5* mutant, we distinguish DTCs that move inward (green lines), anterior DTCs that fail to turn inward (cyan lines) and posterior DTCs that fail to turn inward (magenta lines). **(c)** Sliding average of the D-V position for each DTC in wild-type *qIs56* animals. **(d)**,**(e)** DTC migration velocity *v* as a function of hours after the L3 ecdysis, for (d) A-P migration and (e) D-V migration in wild-type *qIs56* animals. The velocity is given by the time derivative of the sliding averages of DTC position shown in panels (b) and (c). Colored markers indicate migration speed for individual animals, whereas the black line and markers indicate the average over all animals. For A-P migration, $v>0$ corresponds to outward movement and $v<0$ to inward movement. **(f)**-**(h)** A-P migration velocity for the *unc-6;qIs56* mutant. Colors correspond to the different mutant phenotype classes in panel (b). **(i)** Comparison of average A-P migration velocity dynamics between wild-type

(red) and *unc-6* mutant animals (green, cyan and magenta, representing the different migration phenotypes). Anterior DTCs that fail to turn inward (cyan) do not cease A-P movement, whereas posterior DTCS that fail to turn inward (magenta) cease A-P movement to the same extent as DTCs that do turn inward (green).

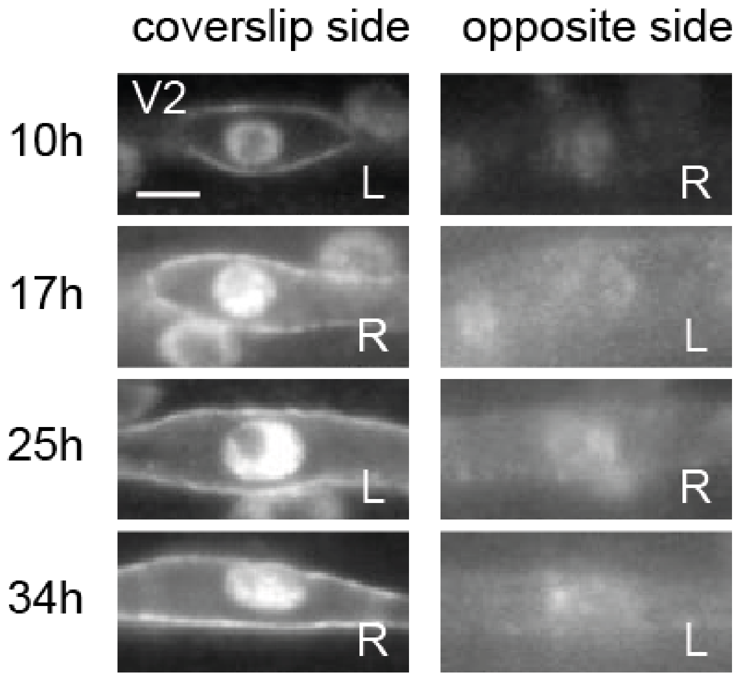

Supplementary Figure 4. Dependence of single-cell fluorescence on cell position

Images of seam cells in the V2 lineage at the time of each *wrt-2* expression peak. Time is hours after hatching. Left column: seam cell closest to the objective. Right Column: corresponding seam cell on the other side of the sample. Scale bar, 5 µm. The label indicated whether the seam cell is on the right (R) or left (L) side of the animal.

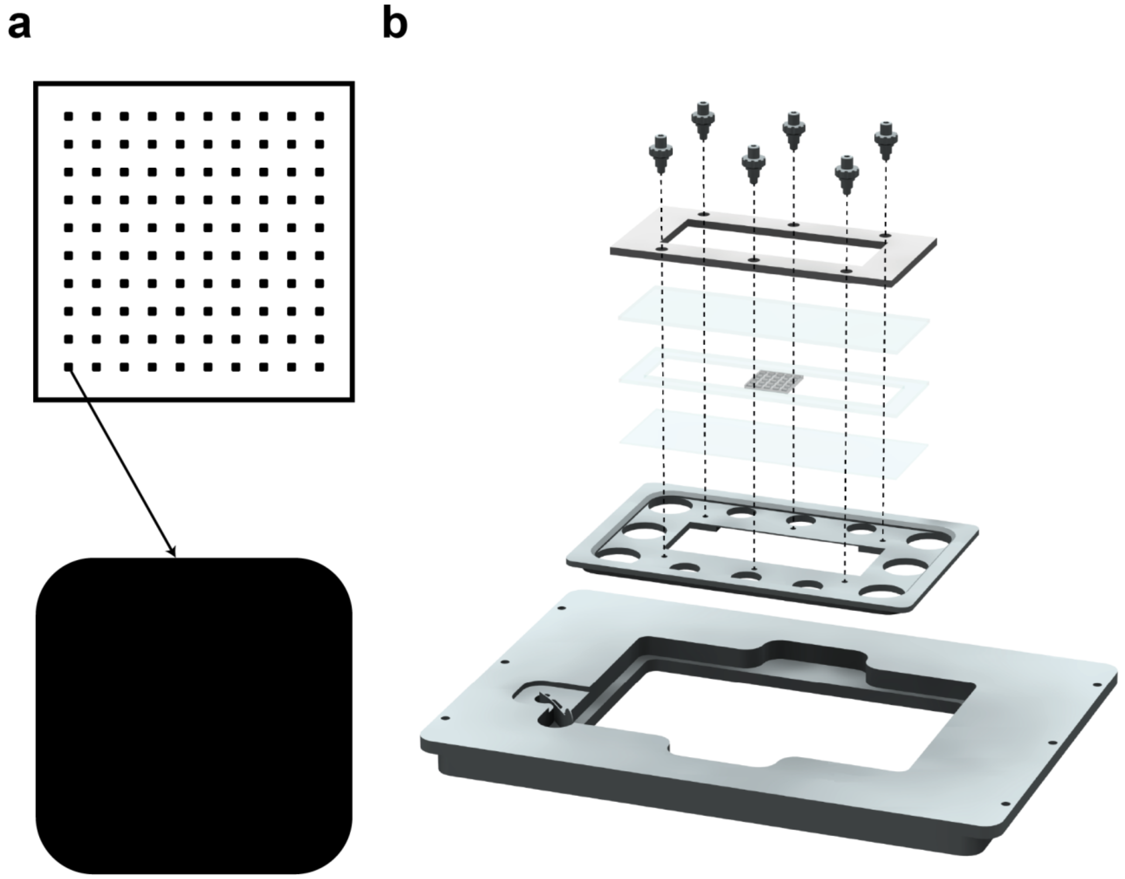

Supplementary Figure 5. Design of microchambers and sample preparation

(a) Design of mask for the microchamber array. For both microchamber dimensions used here (250μm and 290μm), the distance between the centers of adjacent microchambers was 800μm. Microchambers have rounded corners, as sharp corners often trapped the head of the animal, for reasons unknown to us, thereby preventing the animal from feeding. **(b)** Overview of sample holder and sample preparation. The microchambers array (in the center of the image) is sealed in between a coverslip (blue, bottom) and a standard glass slide (blue, top), using a glass spacer with the dimensions of a normal glass slide (blue, middle). Using screws, the sample is then clamped between two metallic holders. To facilitate rapid movement of the piezo Z-stage, the metal parts are made of aluminum and contain holes to reduce their weight. The sample in its holder is then placed on the Z-stage (bottom).

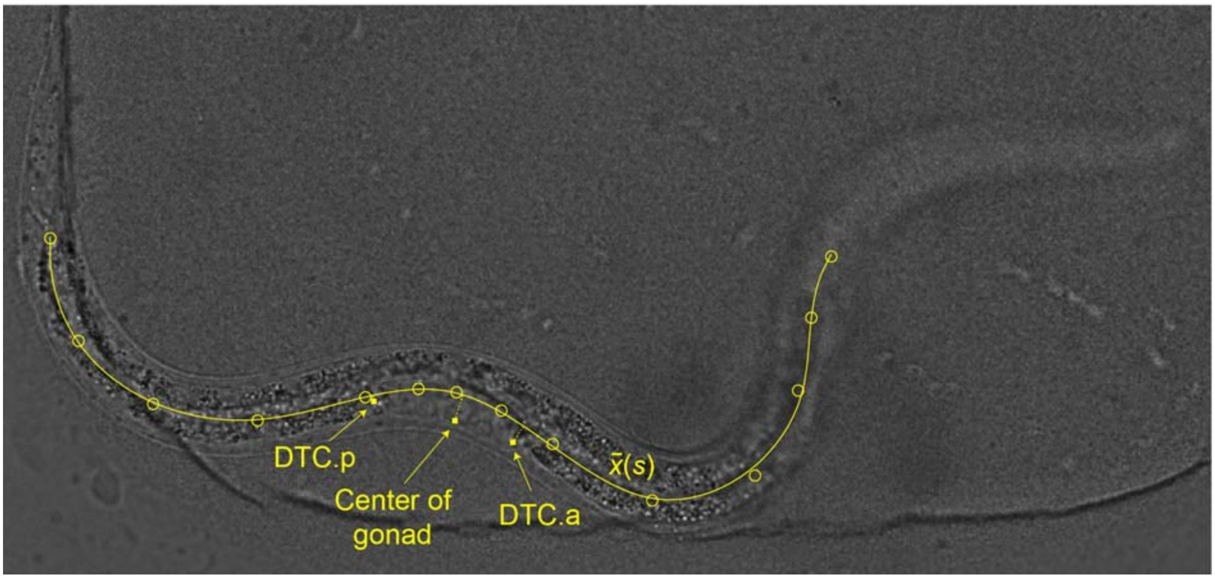

Supplementary Figure 6. Analysis of cell position in body axis coordinates.

Example of annotation of DTC position. Circles show user-selected points that give the animal's body axis, the squares indicate position of the anterior and posterior DTC (DTC.a and DTC.p) as well as the center of the gonad. The line indicates the spline curve $\bar{x}(s)$ fit through the animal's body axis. For each point of interest with image coordinates $\bar{r} = (x, y)$, the A-P position is given by the value of the arc length s_{min} for which the distance $\|\bar{x}(s') - \bar{r}\|$ is minimized (dotted lines). The D-V position is then given by $t = \pm\|\bar{x}(s_{\text{min}}) - \bar{r}\|$, where the sign of t is defined so that for the D-V position of the gonad, $t_{\text{gonad}} < 0$.
